# Natural Products and Antimicrobial Nanoparticles Against Methicillin-Resistant *Staphylococcus aureus*: Mechanisms, Synergistic Interactions, and Therapeutic Potential

**DOI:** 10.3390/pharmaceutics18050515

**Published:** 2026-04-23

**Authors:** Abdulaziz M. Almuzaini, Mahmoud Jaber, Ayman Elbehiry

**Affiliations:** 1 Department of Veterinary Preventive Medicine, College of Veterinary Medicine, Qassim University, Buraydah 51452, Saudi Arabia; 2 Department of Public Health, College of Applied Medical Sciences, Qassim University, P.O. Box 6666, Buraydah 51452, Saudi Arabiaar.elbehiry@qu.edu.sa (A.E.)

**Keywords:** methicillin-resistant *Staphylococcus aureus*, antimicrobial nanoparticles, natural products, biofilm, nanocarriers, synergistic therapy, drug delivery, antibiotic resistance

## Abstract

Methicillin-resistant *Staphylococcus aureus* (MRSA) is a major clinical problem due to its resistance, virulence, and biofilm formation, which diminish antibiotic efficacy. This review explores natural products and antimicrobial nanoparticles (NPs) as alternative and combined strategies for controlling MRSA. Natural compounds, such as plant metabolites, essential oils, antimicrobial peptides, and fungal products, act by disrupting membranes, interfering with cellular processes, and limiting biofilm formation. Antimicrobial NPs, especially metal and metal oxide materials, act through membrane damage, oxidative stress, and metal ion release, enabling activity against resistant bacteria and improving biofilm penetration. Combining natural products with NPs increases stability, delivery, and local activity, enhances antibacterial effects, and reduces effective doses. Green synthesis enables direct integration of bioactive compounds, while nano-delivery platforms optimize solubility and controlled release. Nanotechnology-based applications such as wound dressings, nanocarriers, and multifunctional platforms support localized and sustained treatment and promote tissue repair. Despite these advances, clinical use is still constrained by safety concerns, variability in NP properties, and the lack of standardized evaluation and regulatory frameworks. Overall, combining natural products with antimicrobial NPs offers a practical strategy to augment MRSA treatment, but further progress depends on consistent design, robust safety evaluation, and clinical translation.

## 1. Introduction

Antimicrobial resistance (AMR) is widely recognized as a major threat to global health and clinical practice [1,2,3]. Current estimates indicate that bacterial AMR caused 1.27 million deaths in 2019 and contributed to nearly 4.95 million deaths worldwide [4]. The World Health Organization (WHO) attributes much of this burden to the misuse and overuse of antimicrobial agents in human, animal, and agricultural sectors [2]. Recent analyses also show an uneven distribution of impact, with declining mortality in younger populations but a clear increase among older individuals [5].

The rising incidence of multidrug-resistant infections has intensified this challenge. These infections occur in both healthcare and community settings, with higher rates in hospitals and a steady increase outside clinical environments [6,7]. As a result, many infections are becoming harder to manage, placing sustained pressure on healthcare systems worldwide [4,8].

Among resistant pathogens, *Staphylococcus aureus* (*S. aureus*) continues to be a major concern due to its ability to cause a wide range of infections, comprising skin and soft tissue infections as well as severe conditions such as pneumonia, bacteremia, endocarditis, and infections of bone and joints [9,10,11]. The organism frequently colonizes the skin and nasal cavity in healthy individuals, facilitating transmission and long-term persistence [12]. The methicillin-resistant form, known as methicillin-resistant *Staphylococcus aureus* (MRSA), is established in both hospital and community environments [13,14]. Epidemiological evidence continues to identify MRSA as a persistent global problem affecting diverse populations and healthcare systems [15].

The impact of MRSA extends beyond its high prevalence and includes a measurable contribution to mortality associated with AMR, with more than 100,000 deaths reported globally in 2019 [16]. High levels of colonization and infection in healthcare facilities and long-term care settings further reflect its continued presence in clinical environments [17].

The efficacy of conventional antibiotics against MRSA has decreased over time. Even with multiple agents available, clinical outcomes are frequently constrained by reduced susceptibility and the development of multidrug-resistant strains [13,18]. In clinical practice, this leads to prolonged infections, increased treatment costs, and a higher likelihood of therapeutic failure [15,19]. Persistent infections are often linked to structured bacterial communities that reduce antimicrobial activity and complicate treatment outcomes [20].

These limitations have driven renewed interest in antibacterial agents derived from natural sources. Natural products have played a central role in anti-infective drug discovery and continue to provide a wide range of bioactive compounds [21,22]. These include plant-derived metabolites, microbial products, and antimicrobial peptides, many of which exhibit activity against resistant bacteria [22,23,24,25]. Their structural diversity supports ongoing efforts to identify new therapeutic candidates [20].

In parallel, advances in nanotechnology have enabled the development of antimicrobial materials with distinct functional properties. Nanoparticles (NPs), particularly those based on metals and metal oxides, exhibit strong antibacterial activity linked to their small size and large surface area [26,27]. These features promote interaction with bacterial cells and support their use as alternative antimicrobial agents [26,28].

Recent studies reveal that combining natural products with nanotechnology-based platforms may provide additional benefits. These strategies enhance the stability and delivery of bioactive compounds and may reduce the required therapeutic dose [29,30]. This integrated framework is now considered a promising strategy to address resistant infections and overcome the limitations associated with single-agent therapies [21,30].

Despite extensive research, current approaches remain insufficient to address MRSA persistence, underscoring the need for integrated, mechanism-based strategies. From this perspective, this review critically examines the antibacterial potential of natural products and antimicrobial NPs against MRSA. It integrates individual and combination strategies to advance the management of resistant infections.

Most available reviews treat natural compounds and antimicrobial NPs as distinct strategies, lacking a coherent integrative perspective. In contrast, this review links NP physicochemical properties with the biological activity of natural compounds to explain how combined systems influence membrane integrity, intracellular processes, and biofilm-associated tolerance in MRSA [26,28,31]. It also differentiates between direct antibacterial effects and delivery-related contributions, clarifying how nanocarriers enhance stability and local availability without acting as primary antimicrobial agents [32,33]. To provide a more structured framework, current evidence is organized into three functional categories: standalone activity, delivery-related enhancement, and combined effects that increase antibacterial performance. This organization supports a clearer interpretation of variable findings and helps define practical priorities for future therapeutic development [30,34].

## 2. Molecular Basis of MRSA Pathogenicity and Resistance

### 2.1. Evolution and Epidemiology of MRSA

MRSA emerged soon after the introduction of methicillin and has since developed into a globally distributed pathogen with multiple genetic lineages [14,35,36]. The acquisition of mobile genetic elements, particularly the staphylococcal chromosomal cassette mec (SCCmec), has been a key event in its evolution and diversification [37,38,39].

MRSA is classified into hospital-acquired MRSA (HA-MRSA), community-acquired MRSA (CA-MRSA), and livestock-associated MRSA (LA-MRSA) based on epidemiological origin and transmission patterns [40,41,42,43]. HA-MRSA is linked to healthcare settings and commonly affects patients with underlying conditions. CA-MRSA circulates outside hospitals and can infect healthy individuals [40,41,44]. LA-MRSA originates from animal reservoirs and can be transmitted to humans [42]. The boundaries between these groups have become less distinct, reflecting ongoing transmission between community and healthcare settings [14].

The global distribution of MRSA is shaped by clonal expansion and adaptation to diverse ecological niches [18,45]. This adaptability supports long-term persistence in both healthcare systems and the community and complicates infection control efforts [13].

### 2.2. Molecular Determinants of Methicillin Resistance

Resistance to β-lactam antibiotics is the defining feature of MRSA and is primarily mediated by the *mecA* gene [46,47]. This gene encodes penicillin-binding protein 2a (PBP2a), which has low affinity for β-lactam antibiotics [37,48]. As a result, cell wall synthesis continues even in the presence of these antibiotics [49].

The *mecA* gene is located within SCCmec, a mobile genetic element that enables transfer between staphylococcal strains and drives the spread of resistance [47,50]. Additional genes and regulatory systems influence resistance levels by affecting cell wall synthesis and interactions with antibiotic targets [18,46]. These combined genetic and regulatory features allow MRSA to maintain resistance under different environmental conditions, which compromises the efficacy of β-lactam therapy [51].

### 2.3. Virulence Factors and Host Interaction

The pathogenicity of MRSA depends on a wide range of virulence factors that support colonization, immune evasion, and tissue damage [9]. Surface adhesins promote attachment to host tissues, while secreted toxins disrupt host cells and immune responses [52].

MRSA produces several toxins, such as pore-forming toxins and superantigens, which contribute to tissue injury and systemic inflammation [53]. Adhesins also enable binding to extracellular matrix components, supporting persistence within host tissues [54].

Coordinated regulation of these factors allows MRSA to adapt to different host environments and sustain infection under varying conditions [55]. This adaptability contributes to the severity and diversity of MRSA-associated diseases.

### 2.4. Biofilm Formation and Antibiotic Tolerance

Biofilm formation is a key factor in the persistence of MRSA infections. MRSA forms structured communities that attach to surfaces and are embedded within a self-produced extracellular matrix [56,57]. This matrix contains polysaccharides, proteins, and extracellular DNA that provide structural stability and protection [58,59].

Biofilm development follows defined stages that include initial attachment, accumulation, maturation, and dispersion [60,61,62]. During these stages, bacterial cells undergo physiological changes that support survival under adverse conditions [62,63].

Cells within biofilms show reduced susceptibility to antimicrobial agents due to limited drug penetration, altered metabolic activity, and the presence of persister cells [64,65,66]. This reduced susceptibility contributes to antibiotic tolerance and supports chronic infection [65,67].

**Figure 1:**
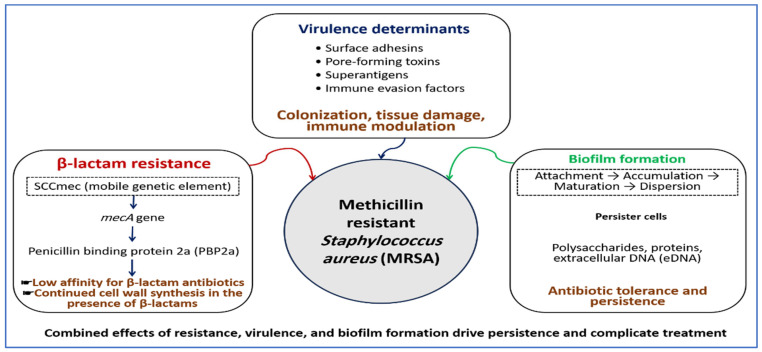
Molecular basis of resistance, virulence, and biofilm formation in MRSA. Resistance to β-lactam antibiotics is mediated by the *mecA* gene located on the SCCmec, which encodes PBP2a with reduced affinity for β-lactams. Virulence determinants, particularly adhesins, toxins, and immune evasion factors, support colonization and tissue damage. Biofilm formation proceeds through attachment, accumulation, maturation, and dispersion, and involves extracellular matrix components such as polysaccharides, proteins, and extracellular DNA, as well as persister cells. These combined mechanisms promote persistence and complicate treatment.

The combined effects of biofilm formation, intrinsic resistance, and virulence factors explain why MRSA infections are difficult to treat and often recur [18,57]. The main mechanisms that contribute to resistance, pathogenicity, and persistence in MRSA are summarized in Figure 1. These interconnected mechanisms create a highly adaptive pathogen that compromises the efficacy of single-target therapies and complicates long-term infection control.

## 3. Natural Products as Antibacterial Agents Against MRSA

Natural products with antibacterial activity against MRSA include a wide range of secondary metabolites derived from plants and microorganisms. These compounds can be broadly classified into major groups such as phenolic compounds, flavonoids, alkaloids, and terpenoids [31,68]. Furthermore, volatile phytochemicals, particularly those found in essential oils (EOs), represent an important subgroup with distinct properties and modes of action [69,70,71]. These volatile compounds often exert antibacterial effects through disruption of bacterial membranes and interference with cellular integrity, thereby complementing the activity of non-volatile metabolites [70,71].

### 3.1. Plant-Derived Secondary Metabolites

Plant secondary metabolites represent a structurally diverse group of compounds with direct antibacterial activity against MRSA [31,68]. Major classes include flavonoids, phenolic compounds, alkaloids, and terpenoids, which target essential cellular processes [31].

Many of these compounds act at the cell envelope, where prenylated flavonoids disrupt membrane integrity and promote leakage of intracellular components, leading to rapid loss of viability [72]. In parallel, several plant metabolites interfere with intracellular enzymatic pathways linked to cell wall synthesis, metabolism, and protein function, which limits bacterial growth [73,74].

Some compounds also affect nucleic acid processes by interfering with DNA replication and transcription, thereby impairing cellular function and reducing bacterial proliferation [73]. Furthermore, plant-derived metabolites can reduce early biofilm development by limiting bacterial adhesion and aggregation, which restricts the formation of stable communities and decreases persistence [75,76].

### 3.2. EOs and Volatile Phytochemicals

EOs are mixtures of volatile plant-derived compounds with strong antibacterial activity against MRSA [69,71]. They are rich in terpenes and phenolic constituents that interact directly with bacterial membranes.

Their activity is primarily linked to rapid disruption of membrane structure and function. Compounds such as thymol and carvacrol increase membrane permeability, leading to leakage of ions and intracellular molecules and loss of cell integrity [70,77]. In addition to structural damage, these compounds alter membrane potential and proton gradients, which reduces intracellular ATP levels and interferes with energy-dependent processes [77].

EOs also affect the early stages of biofilm development. Key components such as thymol and carvacrol reduce bacterial adhesion and significantly decrease biofilm formation, while compounds such as eugenol weaken cell-to-cell interactions and reduce matrix stability [78,79,80]. Compared with other plant-derived compounds, their effects are more closely associated with rapid membrane destabilization and disruption of cellular energetics.

### 3.3. Antimicrobial Peptides and Natural Defense Molecules

Antimicrobial peptides (AMPs) are small bioactive molecules produced by plants, animals, and microorganisms as part of innate defense systems [81,82]. They show strong activity against Gram-positive bacteria, including MRSA [83,84].

Their primary mode of action involves electrostatic interaction with bacterial membranes, where positively charged peptides bind to negatively charged surfaces and induce membrane destabilization and permeabilization, resulting in rapid loss of cellular integrity [85,86]. In contrast to many plant-derived compounds, this interaction is driven by charge-dependent targeting rather than passive diffusion.

Some peptides penetrate the cell and interfere with intracellular components involved in transcription and translation, disrupting essential processes [85,87]. Certain peptide antibiotics bind to ribosomal subunits and inhibit protein synthesis, leading to bacterial death [84,88].

AMPs also limit early biofilm development by interfering with attachment and aggregation, which reduces persistence and prevents the establishment of stable structures [89,90].

### 3.4. Bioactive Compounds from Fungi and Mushrooms

Fungi and mushrooms are important sources of antibacterial metabolites with activity against MRSA [22,91]. These include polyketides, peptides, and terpenoid derivatives with diverse biological functions [92].

Fungal metabolites exhibit greater diversity in their modes of action. Some compounds increase membrane permeability and induce leakage of intracellular components, while others act on intracellular targets [93]. Pleuromutilin antibiotics, for example, bind to the bacterial ribosome and inhibit peptide bond formation, which prevents bacterial growth and replication [94,95].

Additional effects include interference with enzymatic pathways involved in cell division and metabolism, leading to reduced bacterial viability [22,96]. These compounds also affect biofilm development by limiting adhesion and early formation, while certain metabolites disrupt established biofilm architecture and interfere with regulatory pathways [97,98,99,100]. Overall, fungal-derived compounds show a wider range of intracellular and regulatory effects compared with other natural product classes, reflecting their structural diversity and mechanistic diversity.

Although many natural compounds demonstrate strong antibacterial activity under in vitro conditions, their performance in vivo is often less consistent due to limitations in stability, bioavailability, and metabolic degradation [21,22,101]. Differences in experimental design, including strain selection and assay conditions, also contribute to inconsistencies in reported outcomes [31,68]. These factors require careful interpretation of in vitro findings and support the use of delivery strategies to improve stability and effective target exposure [32,33]. Moreover, the contribution of individual antibacterial effects differs among compound classes, and not all reported activities are consistently supported under varying experimental conditions.

**Figure 2:**
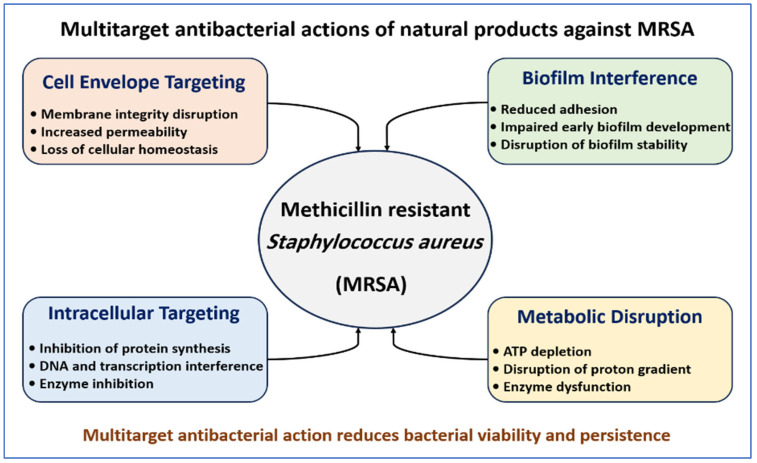
Multitarget antibacterial actions of natural products against MRSA. Natural compounds act through complementary mechanisms including membrane disruption, intracellular interference, metabolic disruption, and inhibition of biofilm development. These combined effects reduce bacterial viability and limit persistence.

Representative natural compounds, their antibacterial mechanisms, and their experimentally validated effects against MRSA are summarized in Table 1. The principal antibacterial targets of natural compounds against MRSA are summarized in Figure 2.

## 4. Antimicrobial NPs as Standalone Antibacterial Agents

Within the framework of this review, nanoparticles are considered here as standalone antibacterial agents, where activity arises from intrinsic physicochemical interactions with bacterial cells [102]. Evaluation of the antibacterial activity of NPs is commonly carried out using standard antimicrobial sensitivity assays. Agar diffusion methods, including disk diffusion and well diffusion tests, are widely used to provide a qualitative assessment based on inhibition zones [27]. Broth-based methods such as minimum inhibitory concentration (MIC) and minimum bactericidal concentration are used for quantitative evaluation of antibacterial activity [27,102].

Additional approaches, including time-kill assays and biofilm inhibition studies, are used to examine bactericidal effects over time and activity against structured bacterial communities [102]. However, differences in experimental conditions, NP dispersion, and interactions with testing media can influence the results. These factors highlight the need for careful interpretation and enhanced standardization of testing protocols [103].

### 4.1. Types of Antimicrobial NPs

Metal and metal oxide NPs represent a major class of antibacterial materials with intrinsic activity against MRSA. The most widely studied systems include silver (Ag), zinc oxide (ZnO), copper oxide (CuO), and titanium dioxide (TiO_2_), all of which exhibit activity against both Gram-positive and Gram-negative bacteria [104,105].

These materials differ in composition, size, surface charge, and reactivity, which determine their interaction with bacterial cells and directly influence antibacterial performance [106]. Unlike conventional antibiotics, their activity does not rely on a single molecular target but arises from combined physicochemical interactions, which reduces the likelihood of resistance development [104].

Other NPs, including gold (Au) and iron oxide (Fe_3_O_4_), also demonstrate antibacterial activity against *S. aureus*. Their antimicrobial efficacy depends strongly on particle size and surface characteristics, making them useful models for understanding structure and activity relationships [28,107].

Particle size is a key determinant of activity, as smaller NPs provide a larger surface area that enhances reactivity and promotes close interaction with bacterial cells [108,109]. Surface charge also plays an important role, with positively charged NPs showing stronger interaction with negatively charged bacterial membranes, which facilitates adhesion and initiates cellular damage [102].

Crystal structure and surface defects further influence reactivity. Metal oxide NPs such as ZnO and TiO_2_ exhibit enhanced antibacterial activity under specific conditions, including light exposure or aqueous environments, due to increased generation of reactive species [110,111]. These physicochemical properties collectively define NP behavior and distinguish them from conventional antibacterial agents. These physicochemical features are particularly relevant in MRSA, where reduced antibiotic susceptibility and adaptive resistance mechanisms require multi-target antibacterial approaches.

### 4.2. Mechanisms of Antibacterial Action

The antibacterial activity of NPs arises from interactions at the nano-bio interface, where multiple processes occur in parallel rather than as isolated events. A central contribution involves the generation of reactive oxygen species (ROS), including superoxide radicals, hydroxyl radicals, and hydrogen peroxide, which induce oxidative damage to lipids, proteins, and nucleic acids and reduce bacterial viability [112,113].

In addition to oxidative stress, NPs establish direct contact with the bacterial surface. Their small size and high surface area enable close interaction with the cell envelope, leading to increased membrane permeability and loss of structural integrity through electrostatic attraction between NP surfaces and bacterial membranes [112,114]. This surface interaction also facilitates entry into bacterial cells [102].

Once internalized, NPs interfere with intracellular components through the combined effects of ROS and released metal ions, which contribute to protein dysfunction and DNA damage and disrupt essential cellular processes [115,116]. Metal ion release, particularly from Ag^+^ and Zn^2+^, further amplifies these effects by binding to functional groups in proteins and enzymes, leading to loss of activity and metabolic imbalance [116].

Understanding these mechanisms requires appropriate analytical and characterization methods [102]. Techniques such as electron microscopy, including scanning electron microscopy (SEM) and transmission electron microscopy (TEM), are used to examine NP morphology and their interaction with bacterial cells [106]. Spectroscopic methods, including ultraviolet–visible spectroscopy (UV-Vis) and Fourier transform infrared spectroscopy (FTIR), provide insight into surface properties and chemical composition [106]. In addition, assays evaluating reactive oxygen species generation, membrane integrity, and intracellular damage support mechanistic interpretation [102,117]. These methods are essential for optimizing NP design and achieving consistent antibacterial performance [117].

In addition to metal oxide NPs, hydroxy double salts (HDS), particularly zinc-based systems such as zinc basic salts, have gained attention as layered inorganic materials with adjustable properties. They are formed from positively charged hydroxide layers separated by exchangeable anions, which allows incorporation of different molecules and control over their behavior [118].

Recent studies show that zinc-based HDS can inhibit bacterial growth when used in composite systems. This effect is linked to the release of Zn^2+^ ions and interactions with bacterial cell surfaces [119]. As well, combining HDS with polymeric matrices or coatings can optimize material stability and support antimicrobial performance [120].

HDS materials have also been explored as delivery platforms. Their structure allows loading of therapeutic agents and gradual release over time, which supports their use in drug delivery applications [118].

Unlike natural compounds that often act through defined molecular targets, NP activity depends strongly on physicochemical properties such as size, surface charge, and environmental conditions, resulting in inconsistent antibacterial outcomes across experimental systems. These mechanisms act together to produce broad-spectrum antibacterial activity, which is particularly relevant for MRSA, where resistance mechanisms compromise the efficacy of single-target therapies.

**Figure 3:**
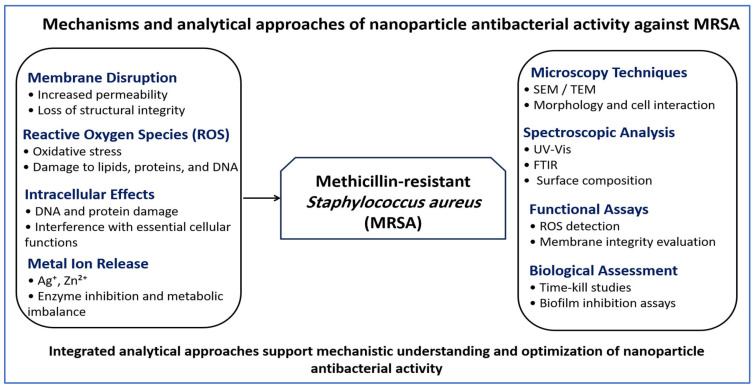
Mechanisms and analytical methods of NP antibacterial activity against MRSA. NPs act through membrane disruption, oxidative stress, intracellular interference, and metal ion release. These effects are evaluated using microscopy, spectroscopic techniques, and functional assays, enabling clear interpretation of antibacterial activity and guiding NP design optimization.

Although several antibacterial effects have been described for NPs, their relative importance is not equivalent. Direct interaction with the bacterial surface, resulting in increased membrane permeability, is consistently observed and represents the primary mechanism, particularly for metal-based NPs [109,113]. In contrast, ROS generation contributes to antibacterial activity but depends strongly on particle composition and environmental conditions [102,103]. Intracellular damage, including effects on proteins and nucleic acids, typically occurs as a secondary consequence of membrane disruption or oxidative stress rather than as an independent pathway [112,113]. These observations establish a functional hierarchy in which membrane interaction is dominant, oxidative stress is condition-dependent, and intracellular effects are largely downstream processes. Figure 3 provides a schematic overview of these mechanisms and their associated analytical methods.

### 4.3. Anti-Biofilm Activity

NPs exhibit strong activity against bacterial biofilms. Their small size enables penetration into the biofilm matrix, allowing access to bacterial cells protected from conventional antimicrobial agents [121]. Within this environment, NPs disrupt matrix structure and reduce bacterial viability through combined physicochemical effects. ROS generation and direct interaction with bacterial cells contribute to structural damage and destabilization of biofilm architecture [121,122]. In this context, NP surface properties govern their interaction with biofilm systems.

Surface modification enhances anti-biofilm performance by regulating interactions with microbial cells and the surrounding matrix. Methods such as coating, encapsulation, and functionalization adjust surface charge and hydrophobicity, influencing adhesion and penetration within biofilms. These features increase penetration through the extracellular polymeric matrix and increase antimicrobial action in structured bacterial communities [123,124]. Tailored surface characteristics also reinforce interactions with bacterial cells and promote disruption of biofilm structure [125].

Surface characteristics further affect NP stability under biological conditions. In complex media, aggregation or surface changes may occur, reducing functional activity. Interactions with biomolecules can result in the formation of a protein corona, which alters surface identity and influences biological behavior, including cellular interaction and antimicrobial performance [126,127]. Controlling these properties is therefore essential to maintain stability and consistent activity in biofilm environments.

NPs also interact with extracellular matrix components, modifying matrix organization and reducing structural cohesion, which enhances penetration and antibacterial efficacy [128,129]. They inhibit early-stage biofilm formation by limiting bacterial adhesion and preventing the development of stable structures [122].

Variations in surface charge and chemistry further influence interactions with bacterial cells and matrix components [102]. Metal ion release supports localized antibacterial effects within the biofilm environment [116]. These mechanisms enable activity against both developing and established biofilms, overcoming diffusion barriers that limit conventional treatments. This is particularly relevant for MRSA, where biofilm-associated tolerance contributes to persistent infection and reduced antibiotic efficacy.

### 4.4. Advantages and Limitations

NPs offer several advantages as antibacterial agents due to their multi-target mode of action. Their physicochemical properties enable simultaneous interactions with bacterial membranes, intracellular components, and biofilm structures, which enhances antibacterial efficacy against MRSA [104]. Their small size and high surface area boost interaction with bacterial cells and support penetration into biofilms, increasing activity under conditions where conventional antibiotics are less effective [121].

NP properties are tunable, as variations in size, shape, and surface characteristics influence antibacterial performance and interactions with biological systems [27,117]. In addition, their multi-target activity reduces susceptibility to resistance development [27].

Despite these advantages, several limitations persist. Antibacterial performance varies with physicochemical properties and environmental conditions, which affects reproducibility [103]. Cytotoxicity is a major concern, as ROS generation and metal ion release may also affect mammalian cells and limit clinical application [130].

Accumulation of NPs in biological systems may lead to long-term toxicity, particularly for non-biodegradable materials [117,131]. Standardization is still a challenge, as differences in synthesis and characterization hinder comparison between studies [103]. In addition, large-scale production is constrained by difficulties in maintaining consistent physicochemical properties, which limits clinical and industrial translation [27]. Addressing these limitations is essential for the safe and effective clinical application of NP-based strategies targeting MRSA infections.

Despite strong antibacterial effects observed in vitro, translation to in vivo systems is limited by toxicity concerns, unpredictable NP behavior, and challenges in dose control [130,131]. Differences in particle size, surface characteristics, and environmental conditions generate heterogeneous biological responses and constrain cross-study comparability [103,117]. In addition, the lack of standardized evaluation methods limits reproducibility and hinders direct assessment of antibacterial performance [103]. These factors indicate that antibacterial activity alone is not sufficient to predict clinical applicability.

## 5. Synergistic Interactions Between Natural Compounds and NPs

Combined antibacterial effects can be interpreted through three functional roles that reflect their contribution to overall activity. The first relates to improved stability and delivery of active compounds through NP systems. The second involves increased bacterial susceptibility under combined conditions. The third focuses on the disruption of biofilm structure together with enhanced penetration of active agents into protected bacterial communities. This framework enables consistent cross-study comparison and supports structured evaluation of combined antibacterial strategies [34,132,133]. In contrast to standalone activity, this section examines NPs as delivery platforms and as components of combined strategies that enhance antibacterial performance [27].

### 5.1. Green Synthesis as an Interface Between Natural Compounds and NPs

Natural compounds contribute to NP formation by acting as both reducing and stabilizing agents. Plant-derived biomolecules, including phenolics, flavonoids, and terpenoids, convert metal ions into NPs while forming an organic layer on the particle surface [134,135]. This process generates particles associated with bioactive compounds that influence stability and subsequent biological interactions [132].

The synthesis process involves sequential reduction, nucleation, growth, and stabilization. During bio-reduction, phytochemicals such as phenolics and flavonoids donate electrons to metal ions (e.g., Ag^+^, Au^3+^), converting them into their zero-valent forms (Ag^0^, Au^0^) [134,135]. Functional groups, particularly hydroxyl and carbonyl moieties, facilitate this redox reaction. The reduced atoms then form initial clusters that act as nucleation centers, followed by controlled growth into NPs [136,137].

Stabilization occurs through the adsorption of biomolecules onto the NP surface, forming a capping layer that prevents aggregation [136]. This layer provides steric and electrostatic stabilization, maintains dispersion, and modulates surface reactivity. Accordingly, the composition of plant extracts governs not only reduction efficiency but also particle size, morphology, and surface characteristics, which directly influence biological performance [138,139].

Functional groups within plant extracts facilitate reduction and generate surface-bound layers that limit aggregation and maintain dispersion through steric and electrostatic effects [136,138,140]. Consequently, green synthesis enables controlled NP formation without harsh chemical conditions [137].

Variations in extract composition and synthesis parameters affect particle size, morphology, and surface chemistry, which in turn influence biological behavior and antibacterial performance [138,139]. Overall, natural compounds function as both chemical mediators of NP formation and regulators of physicochemical properties that define interactions with bacterial systems.

### 5.2. Nano-Delivery of Natural Compounds

NP-based systems overcome key physicochemical limitations of natural compounds, particularly poor stability and low aqueous solubility. Encapsulation protects bioactive molecules from degradation and maintains their activity under biological conditions [32,33].

Nano-formulation strategies include liposomes, polymeric NPs, lipid-based carriers, and nanoemulsions, each defined by distinct structural features and loading mechanisms. Liposomes are composed of phospholipid bilayers that accommodate both hydrophilic and hydrophobic compounds, while polymeric NPs enable controlled release through diffusion or matrix degradation. Lipid-based systems enhance the solubility of poorly water-soluble compounds, and nanoemulsions increase dispersion and stability in aqueous environments [141,142,143].

Association with NPs expands localization at the site of infection and promotes interaction with bacterial cells and infected tissues [144]. Controlled release maintains effective concentrations over time and minimizes frequent dosing [33].

Compatibility with biological systems is governed by NP composition, size, and surface properties. Biocompatible materials such as phospholipids and biodegradable polymers reduce cytotoxic effects and support interaction with biological membranes. Surface characteristics regulate cellular uptake, biodistribution, and immune recognition, which collectively influence therapeutic performance in vivo [141,145]. Localized delivery enables higher concentrations within infected tissues while limiting systemic exposure, improving treatment efficiency [144].

Stability in biological environments is influenced by interactions with proteins and surrounding biomolecules. Exposure to biological fluids leads to the formation of a protein corona, which alters surface identity and affects cellular interaction and distribution [126,127]. Moreover, physiological factors such as pH, ionic strength, and enzymatic activity can modify NP structure and drug release behavior. These processes determine circulation time, targeting efficiency, and overall therapeutic outcome [145].

In this context, NPs mainly regulate the distribution, retention, and release of active compounds within biological systems rather than acting as primary antibacterial agents.

### 5.3. Enhancement of Antibiotic Activity

Antibiotic sensitivity differs between Gram-positive and Gram-negative bacteria due to structural differences in the cell envelope. Gram-positive bacteria such as MRSA lack an outer membrane, allowing more direct access of antimicrobial agents to the cell wall [104]. In contrast, Gram-negative bacteria possess an outer membrane enriched with lipopolysaccharides that restrict antibiotic penetration and contribute to intrinsic resistance [104]. These structural features influence how NPs enhance antibiotic activity. In Gram-positive systems, NPs increase membrane permeability and facilitate intracellular access [102,146]. In Gram-negative bacteria, they can disrupt the outer membrane barrier and augment antibiotic uptake [102,104]. This distinction explains the variation in NP-antibiotic synergy observed among bacterial groups [146].

The combination of NPs with antibiotics alters bacterial susceptibility primarily by improving drug access rather than introducing independent antibacterial mechanisms. Experimental studies report reduced MICs when antibiotics are used with metal NPs such as Ag and ZnO [34,146].

This effect is associated with enhanced penetration of antibiotics into bacterial cells. Interactions at the cell envelope increase permeability, enabling more efficient entry and allowing lower concentrations to achieve comparable effects [146]. Increased intracellular accumulation further supports higher local drug availability without modifying the antibiotic structure [34].

Combinations such as AgNPs with ampicillin or vancomycin show enhanced activity against resistant *S. aureus*, including MRSA, indicating partial restoration of antibiotic efficacy under these conditions [34,147]. Reduced efflux activity has also been reported, contributing to intracellular retention [148]. Overall, NPs influence transport and intracellular distribution, which accounts for the observed reduction in effective dosage.

### 5.4. Anti-Biofilm Synergistic Effects

Combined formulations influence biofilm-associated tolerance by strengthening access to bacterial cells within structured communities. NPs can penetrate the biofilm matrix and distribute associated compounds within regions that are less accessible to conventional treatments [132].

Within the biofilm environment, natural compounds act on bacterial cells, while NPs alter matrix organization and enable more uniform distribution of active agents. This interaction affects structural stability and local exposure conditions, leading to reduced biofilm integrity [133].

Experimental studies report stronger inhibition of biofilm formation and greater disruption of established biofilms when NPs are combined with natural compounds [149,150,151]. These effects occur at both early and mature stages, indicating an impact on formation and persistence [133,152].

**Figure 4:**
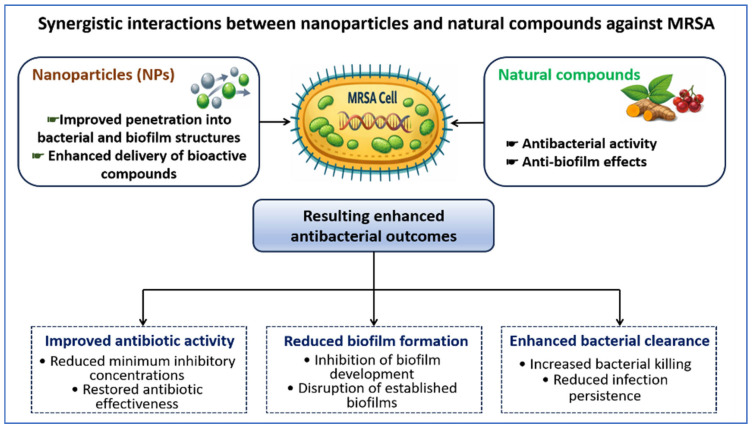
Synergistic interactions between NPs and natural compounds against MRSA. NPs enhance penetration into bacterial and biofilm structures and optimize the delivery of bioactive compounds. Natural compounds provide antibacterial and anti-biofilm activity. Their combination results in potentiated antibiotic activity, reduced biofilm formation, and enhanced bacterial clearance, collectively improving antibacterial efficacy.

Enhanced penetration within the biofilm facilitates disruption of preformed structures that are typically resistant to conventional treatments [152]. The observed outcomes reflect increased accessibility and spatial distribution of active agents rather than the introduction of distinct antibacterial mechanisms (Figure 4).

These effects depend on concentration and exposure duration, which influence both antibacterial activity and biological response. Quantitative toxicity metrics such as half-maximal inhibitory concentration (IC50), lethal concentration (LC50), and effective concentration (EC50) are used to define exposure and response relationships in NP-based systems. Lower values generally indicate higher biological activity, although direct comparison between studies is limited by differences in NP type, test conditions, and biological models. For example, Ivask et al. [153] reported concentration-dependent toxicity profiles for metal oxide NPs and established EC50 values in multiple biological systems, demonstrating that sensitivity varies widely among organisms. This heterogeneity in outcomes reflects differences in particle properties, including size, surface characteristics, and dissolution behavior, as well as experimental design. Subsequently, these metrics can be interpreted within the specific context of each study rather than as universally comparable values [131,154,155].

Although combined NP-based systems often demonstrate enhanced antibacterial effects, meaningful comparison between formulations is limited by heterogeneity in NP composition, synthesis methods, experimental conditions, and reporting practices [103,156]. In many cases, improved outcomes are linked to enhanced delivery or increased local concentration rather than fundamental changes in antibacterial mechanisms [32,144]. Furthermore, most studies report overall effects without distinguishing the contribution of individual mechanisms, which complicates interpretation of dominant pathways. Evidence is largely derived from in vitro models, while in vivo validation is scarce and less consistent, leaving the extent of therapeutic benefit unclear [156,157]. These limitations underscore the need for standardized evaluation methods to achieve robust cross-study comparability of NP-based strategies.

A comparative overview of the main nanotechnology-based antibacterial strategies against MRSA, including their mechanisms, strengths, limitations, and levels of evidence, is presented in Table 2.

## 6. Nanotechnology-Based Applications

Nanotechnology-based platforms provide a practical pathway for translating antibacterial strategies into clinically relevant applications. These formulations are designed to optimize local delivery, stability, and retention of antibacterial agents within infected tissues. Current developments include wound dressings, carrier-based delivery systems, and multifunctional platforms integrating multiple therapeutic roles within a single structure [158]. These strategies are particularly relevant for MRSA infections, where localized and sustained delivery is required to overcome resistance and biofilm-associated persistence.

A clear distinction should be made between experimental findings and clinically validated applications. Most evidence on nanotechnology-based antibacterial strategies against MRSA is derived from in vitro and preclinical studies, whereas clinical data are scarce [156,157]. Accordingly, the following sections examine strategies that have demonstrated experimental promise, although their translation into routine clinical use remains under evaluation.

### 6.1. Wound Dressings

Nanotechnology has been widely applied in the development of advanced wound dressings for infected and chronic wounds. These platforms incorporate NPs into biocompatible materials such as hydrogels and nanofibers to reinforce local antibacterial activity while supporting tissue repair [158].

Hydrogel-based dressings are extensively used due to their high-water content and compatibility with biological tissues. They maintain a moist environment that supports healing and enable controlled release of antibacterial agents in response to local conditions such as pH and enzymatic activity [159].

Incorporation of NPs into hydrogel matrices promotes retention of active agents and supports sustained antibacterial activity. Injectable and sprayable hydrogels further promote clinical applicability by conforming to wound geometry and enabling minimally invasive application [159].

Electrospun nanofiber dressings provide a porous structure with high surface area, allowing efficient drug loading and sustained release. These constructs maintain prolonged contact with the wound surface and enable continuous local delivery of antibacterial agents [145]. Experimental studies demonstrate that nanofiber-based dressings sustain drug release while preserving antibacterial effectiveness, supporting their use in chronic wound management [160].

Overall, nanostructured wound dressings develop infection control and support tissue repair through localized and sustained delivery [158]. This is particularly important in MRSA-associated wound infections, where biofilm formation and delayed healing require prolonged local antibacterial activity.

### 6.2. Nanocarriers

Nanocarrier platforms are widely used to enhance the delivery of antibacterial compounds in both topical and systemic applications. These platforms include liposomes, lipid-based NPs, polymeric NPs, and nanoemulsions [141].

Liposomes are among the most established carriers due to their ability to encapsulate both hydrophilic and hydrophobic compounds. Encapsulation strengthens stability and enables controlled release of therapeutic agents within infected tissues [142].

Nanocarriers enhance retention at the target site and enhance penetration into skin and infected tissues, which reduces systemic exposure and supports more efficient treatment [145].

Polymeric and lipid-based nanocarriers allow adjustment of drug loading and release profiles, enabling sustained delivery under different conditions [143]. These systems are particularly useful for compounds with limited solubility or stability, as they augment availability and prolong activity at the site of infection [145].

Several nanocarrier-based formulations have advanced to preclinical and early clinical evaluation, indicating potential for translation into therapeutic use [141]. Such delivery platforms are especially valuable for MRSA treatment, where effective intracellular and biofilm penetration is required to achieve therapeutic outcomes.

### 6.3. Multifunctional Platforms

Multifunctional platforms are designed to combine antibacterial activity, controlled delivery, and tissue-supporting functions within a single integrated platform to address the complexity of infected wounds [161].

Hybrid materials that incorporate NPs into hydrogel, polymeric, or lipid-based matrices enable simultaneous delivery of antibacterial agents and molecules that promote tissue repair, allowing coordinated treatment within one system [162].

Recent studies show that multifunctional hydrogel-based systems maintain sustained release of therapeutic agents while preserving antibacterial activity in infected wound models [163]. This controlled delivery sustains prolonged local exposure without repeated application.

In addition to infection control, these platforms promote tissue repair. Experimental models demonstrate boosted wound closure, increased collagen deposition, and enhanced vascular development in MRSA-infected wounds treated with NP-integrated systems [164]. These effects are particularly relevant in chronic wounds, where infection and delayed healing occur simultaneously.

Lipid-based multifunctional platforms further increase retention of therapeutic agents within the wound environment while promoting tissue recovery [142]. Responsive platforms have also been developed to regulate release according to local conditions such as inflammation or oxidative stress, enhancing targeting and adaptability throughout the wound healing process [163].

Although many multifunctional platforms are still under investigation, current evidence indicates a transition toward integrated therapeutic platforms that combine antibacterial activity, controlled delivery, and tissue repair within a single adaptable system [165]. These integrated strategies are particularly suited for MRSA management, where infection control and tissue regeneration must be addressed simultaneously.

Overall, despite strong experimental antibacterial performance, few strategies have progressed to clinical application, underscoring ongoing challenges in translation and validation [156,157].

## 7. Safety and Regulatory Considerations

The translation of nanotechnology-based antibacterial platforms into clinical use requires careful evaluation of safety, dose, environmental impact, and regulatory feasibility. Although these systems offer clear therapeutic potential, their broader application is constrained by toxicity concerns, long-term exposure risks, and the lack of fully established regulatory frameworks [166,167,168]. These challenges are particularly relevant to MRSA-targeted therapies, where effective treatment often requires higher local concentrations and prolonged exposure.

Toxicity assessment of NP-based systems depends on dose, exposure duration, and physicochemical characteristics such as size, surface charge, and composition. Nonetheless, defining consistent safety thresholds is challenging due to variability in experimental models and testing conditions. Differences in cell types, assay methods, and exposure protocols often produce inconsistent results, which limits direct comparison between studies. This lack of standardization complicates the interpretation of safety data and hinders the establishment of reliable safety margins.

### 7.1. Cytotoxicity and Biological Safety

Cytotoxicity persists as a central concern in the biomedical use of NPs. Experimental evidence shows that exposure can reduce cell viability and induce cellular damage, including DNA injury, inflammatory responses, and programmed cell death [131,154,155].

Toxic effects are influenced by physicochemical properties such as size, composition, and surface characteristics, as well as exposure conditions. Smaller particles and highly reactive surfaces interact more readily with cellular systems, increasing the likelihood of adverse effects [131,169].

Systemic exposure has also been associated with effects on multiple organ systems, particularly the respiratory and immune systems, under repeated or prolonged exposure conditions [169,170]. These findings highlight the need for comprehensive safety evaluation prior to clinical application. This is particularly important for MRSA infections, where prolonged or repeated treatment may be required to address persistent and biofilm-associated infections.

### 7.2. Dose and Exposure Considerations

Dose plays a central role in determining both therapeutic outcome and toxicity. Available evidence indicates that biological responses to NPs are concentration-dependent, with higher exposure levels associated with increased cellular damage and inflammatory activity [131,169].

Accurate dose control is challenging due to variability in particle size, aggregation behavior, and distribution within biological systems. These factors influence bioavailability and complicate the definition of safe and effective exposure ranges [155,171].

NPs may also accumulate in tissues over time, leading to sustained exposure even at low administered doses. This raises concerns regarding long-term safety and emphasizes the need for careful dose optimization in clinical settings [169,171]. Such considerations are critical in MRSA treatment, where insufficient dosing may fail to eradicate infection, while excessive exposure increases the risk of toxicity.

### 7.3. Environmental Risks

The expanding use of nanomaterials has raised concerns about their release into the environment during production, application, and disposal. Studies indicate that NPs can persist in soil, water, and air, where they undergo transformations that influence their behavior and potential toxicity [172].

Environmental exposure may lead to bioaccumulation in living organisms and affect ecological systems at multiple levels. Reported effects include alterations in growth, metabolism, and reproductive processes in plants and aquatic species [173].

Their small size enables transfer between trophic levels, raising concerns about long-term ecological impact and movement through food chains [174]. These uncertainties highlight the need for comprehensive environmental risk assessment and monitoring.

### 7.4. Regulatory Challenges

Regulatory frameworks for nanotechnology-based platforms are still evolving and lack full standardization. A key limitation is the absence of consistent methods for evaluating toxicity, exposure, and long-term safety among different nanomaterials [175]. Risk assessment is further complicated by the diversity of NP properties, which limits the use of uniform evaluation criteria. Additionally, the cost and complexity of safety testing restrict the availability of comprehensive data required for regulatory approval [176]. Existing regulatory efforts largely adapt conventional guidelines instead of developing NP-specific frameworks. Nevertheless, the distinct behavior of nanomaterials requires dedicated assessment strategies that consider their physicochemical properties and biological interactions [177].

From a regulatory perspective, clinical translation requires consistent characterization of NP properties, reproducible manufacturing processes, and well-defined quality control measures. Differences in formulation and performance are a major challenge, as regulatory evaluation depends on standardized and comparable data. In addition to safety evaluation, quality control is essential for regulatory approval and commercialization of NP-based systems. This requires accurate assessment of particle size, surface characteristics, composition, and stability, together with consistent performance within production batches. Variations in synthesis or formulation can alter biological behavior and complicate regulatory evaluation. Standardized production methods, validated analytical techniques, and transparent reporting of physicochemical and biological properties are therefore necessary to ensure reliability and support regulatory acceptance [175,176,177]. Long-term safety and biological interactions should be carefully evaluated, particularly for systems intended for repeated or prolonged use.

Progress toward clinical implementation depends on improved standardization, robust safety evaluation, and the development of clear regulatory pathways that enable safe and effective use. Establishing such frameworks is crucial for the clinical translation of NP-based therapies targeting MRSA. Key safety considerations affecting clinical translation, including cytotoxicity, dose-related variability, environmental impact, and regulatory challenges, are summarized in Table 3.

## 8. Future Perspectives

Advancing the use of natural products and antimicrobial NPs against MRSA requires a more coherent research framework that addresses current limitations in design, evaluation, and clinical translation. Despite extensive experimental progress, several unresolved issues continue to limit practical application.

A central challenge is the absence of consistent standards for NP production, characterization, and biological testing. Differences in particle size, surface features, and experimental conditions drive inconsistent outcomes and limit cross-study comparability [125]. Establishing unified protocols and reporting criteria will be essential to help reproducibility and enable reliable assessment of antibacterial performance and safety.

Another important limitation relates to the gap between experimental findings and clinical applicability. While many systems demonstrate strong activity under controlled conditions, their behavior in living systems is insufficiently defined [156,157]. Future work should emphasize robust in vivo models, extended safety evaluation, and detailed pharmacokinetic profiling to clarify distribution, retention, and potential adverse effects.

Scalability and manufacturing consistency are still unresolved. Many production methods are difficult to reproduce at scale, and batch-to-batch variability affects both physicochemical properties and biological responses. [178]. Developing production methods that ensure uniformity while remaining technically and economically feasible is essential for clinical implementation.

The capacity of MRSA to adapt to NP-based interventions requires closer investigation. Although these systems act through multiple pathways, bacterial responses such as biofilm reinforcement, efflux activity, and stress adaptation may reduce long-term efficiency [179]. Future studies should examine these responses under clinically relevant conditions and evaluate combination strategies that limit adaptive survival [180].

Biofilm-associated tolerance continues to pose a major barrier to treatment. Current approaches often fail to fully address the structural complexity and physiological heterogeneity of biofilms. Future efforts should focus on enhancing penetration, disrupting matrix organization, and targeting persistent cell populations to achieve more consistent therapeutic responses.

Emerging nanotechnological platforms enable more precise intervention. Responsive delivery systems that adjust to local conditions can increase site-specific activity and reduce unintended exposure [125]. Coupling such platforms with diagnostic tools may support the selection of appropriate treatment strategies based on infection characteristics, improving therapeutic efficiency.

Overall, progress in this field depends on coordinated efforts to enhance methodological consistency, reinforce biological validation, ensure reliable production, and develop targeted delivery systems. Addressing these priorities will be critical for translating current advances into effective clinical strategies against MRSA.

## 9. Conclusions

MRSA is difficult to treat due to resistance, virulence, and biofilm formation, which reduce antibiotic efficacy. Natural products provide bioactive compounds that disrupt membranes, interfere with cellular processes, and limit biofilm development. Antimicrobial NPs act through physicochemical interactions, including membrane disruption, oxidative stress, and metal ion release, enabling activity against resistant bacteria and improving penetration into biofilms. Their combination with natural compounds improves stability, supports delivery, and increases local effectiveness while reducing required doses. Nanotechnology-based platforms, including wound dressings and nanocarriers, enable localized and controlled treatment and support tissue repair. Nevertheless, clinical translation is constrained by safety concerns, differences in nanoparticle properties, and the lack of standardized regulatory frameworks. Despite substantial progress, the field is still limited by variability in experimental design, inconsistent reporting, and insufficient in vivo validation. Addressing these issues is essential to enable reliable comparison between NP-based strategies and to support their clinical application. This review proposes a structured perspective that distinguishes NPs as standalone antibacterial agents, delivery systems, and components of synergistic strategies. This framework enables comparison of different strategies and supports the development of standardized evaluation methods to advance clinical translation.

## Figures and Tables

**Table 1:** Representative natural compounds with validated antibacterial activity against MRSA, highlighting their primary mechanisms of action and corresponding antibacterial consequences.

Source	Compound	Validated Mechanism	Observed Effect (MRSA)	References
Plant	Prenylated flavonoids	Membrane permeabilization	Leakage of intracellular contents; cell death	[72]
EOs	Thymol	Membrane disruption; permeability increase	Reduced viability; ion leakage	[70,77]
	Carvacrol	Membrane permeabilization; ATP depletion	Loss of membrane integrity; metabolic disruption	[70,78]
	Eugenol	Disruption of adhesion and cell signaling	Reduced biofilm formation and stability	[79,80]
AMPs	Defensins	Membrane permeabilization	Rapid bacterial killing	[85,86]
	Ribosome-targeting peptides	Inhibition of protein synthesis	Blocked translation; bacterial death	[84,88]
Fungal	Pleuromutilins	Ribosomal inhibition (peptidyl transferase center)	Inhibition of bacterial growth	[94,95]
	Endophytic fungal metabolites	Increased membrane permeability	Leakage of DNA/proteins; cell death	[93]
	Secalonic acids/ Cytochalasins	Inhibition of biofilm formation and adhesion	Reduced MRSA biofilm development	[98,99,100]

**Table 2:** Comparative overview of nanotechnology-based antibacterial strategies against MRSA.

System Type	Key Components	Primary Mode of Action	Strengths	Limitations	Level of Evidence
Natural compounds	Curcumin, flavonoids, phenolic compounds	Disrupt membrane integrity and interfere with essential cellular processes	Multiple biological targets with generally favorable safety profiles	Limited stability and low availability at the target site	Predominantly in vitro with limited in vivo validation
Metal-based NPs	AgNPs and ZnO NPs	Damage cell membranes and induce oxidative stress through reactive species	Strong antibacterial effect among resistant strains	Potential toxicity and variability linked to particle properties	Supported by both in vitro and in vivo studies
Nanocarrier systems	Liposomes, polymeric NPs, nanoemulsions	Enhance delivery, retention, and distribution within infected tissues	Increased bioavailability and more efficient localization	Formulation complexity and challenges in large-scale production	Mainly preclinical with emerging clinical evaluation
Combined platforms	NPs integrated with natural compounds	Enhance local concentration and facilitate penetration into biofilms	Promoted antibacterial performance and broader functional activity	Limited standardization and unclear contribution of individual components	Largely preclinical

**Table 3:** Safety considerations and regulatory challenges of NP-based antibacterial systems.

Aspect	Key Concern	Impact on Application	References
Cytotoxicity	Cellular damage, inflammation, and reduced viability associated with NP exposure	Limits clinical use and requires careful safety assessment	[131,155]
Dose and exposure	Dose-dependent toxicity and challenges in defining safe exposure levels	Complicates determination of therapeutic dose ranges	[131,169]
Environmental risks	Persistence, transformation, and bioaccumulation in environmental systems	Raises concerns regarding ecological impact and long-term exposure	[172,174]
Regulatory challenges	Lack of standardized evaluation methods and NP-specific guidelines	Delays clinical translation and complicates approval processes	[175,176]

## Data Availability

No new data were created or analyzed in this study.
